# Early Diagnosis of Bronchopulmonary Dysplasia with E-Nose: A Pilot Study in Preterm Infants

**DOI:** 10.3390/s24196282

**Published:** 2024-09-28

**Authors:** Laura Tenero, Michele Piazza, Marco Sandri, Giuliana Ferrante, Elisabetta Giacomello, Benjamim Ficial, Marco Zaffanello, Paolo Biban, Giorgio Piacentini

**Affiliations:** 1 Pediatric Section, Azienda Ospedaliera Universitaria Integrata Verona, 37126 Verona, Italy; laura.tenero@aovr.veneto.it; 2 Department of Surgery, Dentistry, Paediatrics and Gynaecology, University of Verona, 37129 Verona, Italy; michele.piazza@univr.it (M.P.); giuliana.ferrante@univr.it (G.F.); elisabetta.giacomello@studenti.univr.it (E.G.); giorgio.piacentini@univr.it (G.P.); 3 Neonatal Intensive Care Unit, Azienda Ospedaliera Universitaria Integrata Verona, 37126 Verona, Italy; benjamim.ficial@aovr.veneto.it (B.F.); paolo.biban@aovr.veneto.it (P.B.)

**Keywords:** electronic nose, volatile organic compounds, preterms, bronchopulmonary dysplasia, mechanical ventilation, breathomics

## Abstract

Bronchopulmonary dysplasia (BPD) is the most common respiratory disease in preterm and is still associated with increased mortality and morbidity. The great interest lies in identifying early biomarkers that can predict the development of BPD. This pilot study explores the potential of e-nose for the early identification of BPD risk in premature infants by analyzing volatile organic compounds (VOCs) in the exhaled breath condensate (EBC). Fourteen mechanically ventilated very preterm infants were included in this study. The clinical parameters and EBC were collected within the first 24 h of life. The discriminative ability of breath prints between preterms who did and did not develop BPD was investigated using pattern recognition, a machine learning algorithm, and standard statistical methods. We found that e-nose probes can significantly predict the outcome of “no-BPD” vs. “BPD”. Specifically, a subset of probes (S18, S24, S14, and S6) were found to be significantly predictive, with an AUC of 0.87, 0.89, 0.82, 0.8, and *p* = 0.019, 0.009, 0.043, 0.047, respectively. The e-nose is an easy-to-use, handheld, non-invasive electronic device that quickly samples breath. Our preliminary study has shown that it has the potential for early prediction of BPD in preterms.

## 1. Introduction

Bronchopulmonary dysplasia (BPD) remains the most significant respiratory disease among preterm neonates. Despite advances in pre and postnatal care, BPD continues to be associated with increased mortality rates and long-term morbidity, exerting a considerable economic burden on health services [1,2,3].

The etiology of BPD is multifactorial, with pre and postnatal factors contributing to its pathogenesis: preterm birth, genetic predisposition, prenatal infection and inflammation, mechanical ventilation, oxygen toxicity, patent ductus arteriosus, and postnatal infection [4,5].

Since its first description in 1967 by Northway et al., the clinical picture of BPD has changed due to significant advances in care (e.g., antenatal steroids, surfactant) [6]. In the pre-surfactant era, the so-called “old” BPD was the result of severe lung injury caused by aggressive mechanical ventilation and high oxygen supplementation in relatively mature preterm infants. BPD was defined by the presence of persistent respiratory signs and symptoms, the need for supplemental oxygen to treat hypoxemia, and an abnormal chest radiograph at 36 weeks of postmenstrual age [7].

After the introduction of surfactant, with the improved survival of extremely premature neonates, the clinical picture of BPD changed. The so-called “new” BPD often develops in preterm neonates in the canalicular or saccular stage of lung development, at least in the first postnatal days. An imbalance between pulmonary inflammation and lung repair seems to play a key role in the pathogenesis of the “new” BPD [8,9,10], characterized by abnormal lung development or arrest in lung maturation.

Currently, the diagnostic criteria for BPD include treatment with oxygen above 21% for at least 28 days and an assessment of the type of respiratory support at 36 weeks of postmenstrual age (PMA) to define the severity of BPD [11].

Despite recent advances in perinatal care (such as the introduction of prenatal steroid use, surfactant treatment, a shift towards non-invasive ventilation as a first-line form of respiratory support, improved nutrition, etc.), which have led to the increased survival of very preterm babies, the overall incidence of BPD has remained unchanged over the past decade. For babies born before 32 weeks, also known as very preterm infants, the incidence of BPD can vary significantly between different medical centers, ranging from 11 to 50% in relation to a decreasing gestational age and birth weight. Additional risk factors include intrauterine growth restriction, sepsis, and prolonged mechanical ventilation exposure and supplemental oxygen exposure [2,12,13].

At present, there is no specific and effective treatment for BPD and the current management of neonates at risk of BPD is based on strategies to prevent and/or minimize lung damage (e.g., a preference for non-invasive ventilation, targeting lower oxygen saturation levels, glucocorticoids to allow earlier extubation, vitamin A supplementation) [9,14].

The early identification of neonates who will later develop BPD is of paramount importance. In fact, it could enable intervention at an early disease stage, when it may be potentially more effective while avoiding unnecessary exposure to potentially hazardous therapies in infants not at risk. Moreover, it could provide new insights into the pathophysiology of BPD and facilitate the development of new therapies [15].

In recent years, following the development of “omic” sciences to evaluate metabolites and gene and protein expression, researchers have focused their studies on identifying biochemical markers in different matrices to predict infants who will go on to develop BPD [4].

Some studies analyzed cytokines and surfactant components in the lung fluid, exhaled breath or tracheal aspirates, and exhaled temperature to identify potential biomarkers. Although different biomarkers were proposed, none have been validated for clinical use.

Exhaled breath condensate (EBC) is a relatively easily sampled biofluid that provides a non-invasive identification of lung disease. EBC contains aerosolized airway lining fluid that is similar to the lining liquid of the airways and the volatile organic compounds (VOCs) [16].

VOCs are chemical substances produced during both physiological and pathophysiological processes and can be obtained non-invasively from different matrices (e.g., feces, sweat, exhaled breath condensate, urine, etc.). Numerous studies during the last decades evaluated the potential role of VOCs in the diagnosis of several metabolic, malignant, infectious, and inflammatory diseases [17].

Several VOCs contained in the EBC derive from hydrocarbons and are the result of metabolic processes that can alter ongoing pathological and inflammatory processes. Therefore, they could potentially be ideal biomarkers of BPD [18].

VOCs can be analyzed using various technologies, including the electronic nose (e-nose), which is an innovative biomimetic device that simulates the olfactory system. The e-nose was previously used to evaluate various pulmonary pathologies in adult subjects, such as asthma, chronic obstructive pulmonary disease, respiratory infections, lung cancer, and obstructive sleep apnea syndrome [19,20]. The analysis of volatile organic compounds (VOCs) in exhaled breath (EBC) is helpful in diagnosing and monitoring respiratory diseases in children. The changes in resistance of each of the sensors were recorded and collected in an onboard database for generating a distribution (smellprint) that describes the VOC mixture and that can be used for pattern recognition algorithms. The measurement of metabolomic profiles may have important advantages over detecting single markers.

To the best of our knowledge, this study is one of very few to report on the use of e-nose in neonates.

The objective of this pilot study is to evaluate whether the e-nose technology allows for the early identification of premature infants who will later develop BPD by analyzing a VOC profile in the EBC.

## 2. Materials and Methods

### 2.1. Study Design and Participants

The study sample was composed of 14 extremely low gestational age (≤31 weeks) infants (8 females and 6 males) admitted to the Neonatal Intensive Care Unit, Azienda Ospedaliera Universitaria Integrata of Verona, Italy.

This study was performed in accordance with the Declaration of Helsinki. This human study was approved by “Comitato Etico per la sperimentazione clinica di Verona (Italy)”—approval: CESC37. This study was not registered as a clinical trial because it was a pilot study.

All parents, guardians, or next of kin provided written informed consent for the minors to participate in this study.

Infants born at a gestational age of ≤31 weeks of either gender and who required intubation and mechanical ventilation were eligible to participate in this study. Exclusion criteria included not intubated infants, congenital heart diseases, respiratory tract congenital malformations/diseases (cystic fibrosis, adenoid cystic pulmonary malformations, esophageal atresia, diaphragmatic hernia), genetic disorders, and other major congenital malformations.

Infants diagnosed with BPD, defined as supplemental oxygen treatment for at least 28 days of life and requiring positive pressure support and/or receiving oxygen at 36 weeks postmenstrual age (PMA), were allocated to the BPD group. The remaining infants without BPD served as a control group. None presented pulmonary hypertension (PH).

A clinical examination and collection of EBC for e-nose VOC profiling were performed during the first 24 h of life. In addition, data about mechanical ventilation, supplementary oxygen requirements, postnatal infection/sepsis, medication use, and feeding pattern were prospectively collected for each included infant.

### 2.2. Exhaled Breath Condensate (EBC)

EBC from infants on conventional mechanical ventilation was collected within the first 24 h of life. EBC samples were collected from the expiratory limb of the ventilator circuit via a valve-less connector. The sample collection adhered to the guidelines set by the European Respiratory Society and the American Thoracic Society (ATS/ERS) [17], which outline the method for collecting EBC in adult and pediatric patients over 3–4 years of age.

However, no guidelines exist for a sample collection in intubated and ventilated newborns. Consequently, we adapted the European guidelines to this particular category of patients.

To ensure the integrity of samples and the standardization of the procedure, the condensate collector was kept inside a temperature-controlled condenser, as illustrated by Hunt [16]. A specific ventilation circuit (RT225, Fisher and Paykel, Panmure, Auckland, New Zealand) was utilized, equipped with a collection chamber for EBC that was integrated with the expiratory phase of the circuit itself. This setup ensures adequate and constant pressure and oxygenation as imposed by the ventilator. The sample integrity was further preserved by placing the EBC cooling trap in an ice bath and applying it to the container dedicated to EBC collection, as illustrated by Hunt and Kononikhin [16,21,22].

The collection process lasted at least four to five hours for each patient, yielding approximately 5 mL of condensate. The samples were then promptly stored in aliquots within polypropylene tubes at −80 °C at the Medical Research Laboratory of the University of Verona, Pediatrics section. The samples were kept in this location until e-nose analysis. For each patient and each e-nose probe, three replicated measurements were obtained.

### 2.3. Electronic Nose Analysis

EBC samples were analyzed with a commercial e-nose (Cyranose 320; Smith Detections, Pasadena, CA, USA) using a nanocomposite array of 32 organic polymer sensors.

Sensor arrays have been shown to exhibit high sensitivity and selectivity in identifying various analytes. In particular, the use of nanostructured materials such as carbon make gas identifiable at low concentrations [23].

When the sensors are exposed to a mixture of VOCs, the polymers swell, thus inducing a change in their electrical resistance.

The measure was performed through an analysis of the VOCs present in the headspace of the tube. To ensure homogeneous study conditions, samples were thawed to room temperature, then maintained in a bath at 37 °C for 30 min to increase the concentration of headspace VOCs and then analyzed in random order. In order to standardize the measurements obtained, we performed a purge of the sensors aimed at removing any remaining VOCs. This washout corresponds to 1.5 times (90 s) more than the analysis time (60 s), as the method described by Visser and colleagues [24]. Moreover, to standardize measurements, we performed three replicates of the analysis for each sample. The values of each probe were standardized, and all the subsequent statistical analyses were performed on the mean values of the standardized replicates.

The changes in resistance of each of the 32 sensors were recorded (and collected) in an onboard database and analyzed by machine learning methods: the distribution pattern generated, or smell-print, describes the VOC mixture.

### 2.4. Statistical Analysis

All subsequent statistical analyses were performed using the means derived from the three observed replicates. The values for each of the 32 probes across the 14 patients were standardized by subtracting the mean and dividing by the standard deviation. Continuous variables were presented as the mean ± standard deviation (SD), and the significance of differences between the mean values of two independent samples was assessed using the two-tailed Student’s *t*-test. Categorical variables were summarized using frequency counts and percentages, with differences between proportions evaluated using Fisher’s exact test. A *p*-value of <0.05 was considered statistically significant.

The potential of the 32 e-nose nanosensors to discriminate between the two study groups was initially explored using partial least squares discriminant analysis (PLS-DA), a pattern recognition method for classification [25]. This approach is particularly advantageous when handling datasets with numerous variables and relatively few samples. The predictive accuracy of PLS-DA was evaluated through repeated cross-validation, which involves, at each repetition, splitting the data into training and testing sets, building the model on the training sets, and testing its ability to correctly classify the observations in the testing sets.

Two different methods were used to evaluate the discriminative ability of each nanoprobe: the estimation of variable importance with the use of a gradient-boosting machine [26] and the application of exact logistic regression [27]. The performance of predictive models was evaluated by calculating the area under the receiver operating characteristic curve (AUC).

The responses of the most predictive nanosensors were further analyzed using a principal component analysis (PCA) with varimax rotation. The e-nose patterns of the study patients were visualized on a plane using the first two principal scores, and a separating line estimated by logistic regression was drawn to discriminate the two groups. Additionally, a classification algorithm based on random forests [28] was implemented using the selected nanosensors as predictors.

The data were analyzed using Stata software (Stata-Corp. 2017. Stata Statistical Software: Release 16. College Station, TX, USA: StataCorp LLC) and R version 4.4.1 (R Core Team (2021). Vienna, Austria. https://cran.r-project.org/, access date 25 September 2024).

## 3. Results

### 3.1. Patient Population

EBC samples were collected within the first 24 h after birth from 14 mechanically ventilated very-low-birth-weight (VLBW) infants. They were subsequently categorized into two groups: those who did not develop BPD (*n* = 5), and those who later developed BPD (*n* = 9). The group of infants that developed BPD, compared to the control group, had a lower gestational age (26.1 vs. 28.3 weeks) and weight (894 g vs. 1182 g). These differences were not statistically significant (Table 1).

All preterm infants included in this study were intubated and mechanically ventilated with/without oxygen supplementation in the first 48 h of life. Additional characteristics that have clinical relevance for BPD development, are reported in Table 1.

### 3.2. Exhaled Breath VOC by E-Nose Analysis

**Figure 1:**
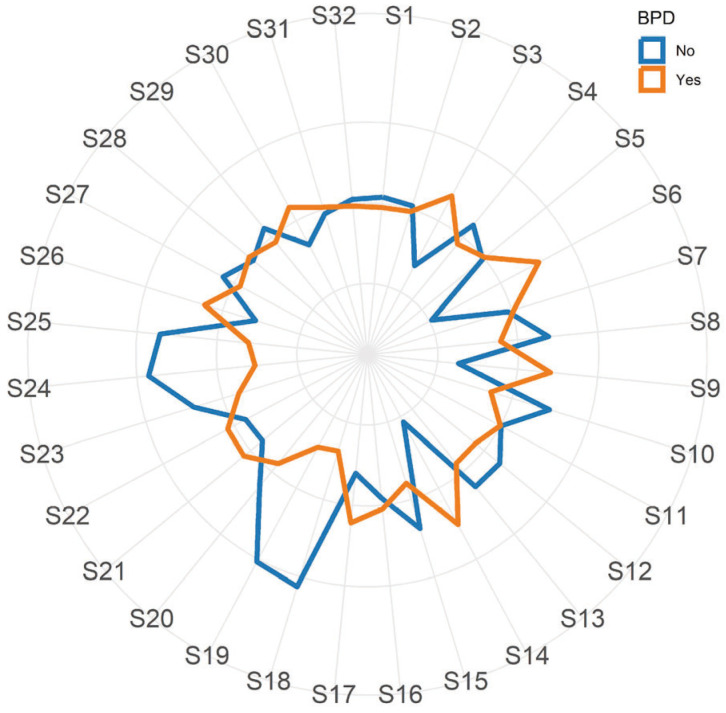
Radar plot depicting mean values of the standardized responses from the 32 e-nose nanosensors in patients who will (orange line) and will not (blue line) later develop bronchopulmonary dysplasia (BPD).

In Figure 1, the standardized e-nose responses for patients who will and will not later develop BPD are depicted, illustrating the average profiles. A visual examination of Figure 1 reveals noticeable variations between the two profiles for probes S6, S14, S18, S19, and S24. These observations suggest that the e-nose has the potential to distinguish between individuals belonging to the two groups.

**Figure 2:**
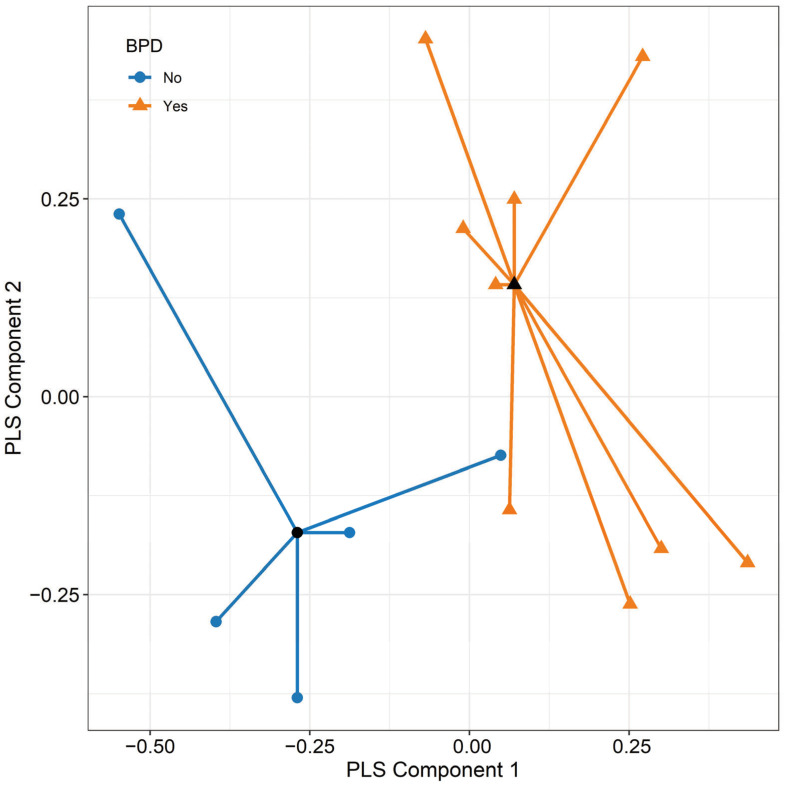
Scatterplot representing electronic nose data, projected onto the subspace identified by the first two components of partial least squares discriminant analysis (PLS-DA). The plot depicts patients who will later develop BPD as orange points and those who will not as blue points. Black dots indicate the mean values of the components for each group.

The overall discriminative power of the e-nose data was assessed using PLS-DA. Figure 2 depicts the projection of the 32 nanoprobes for the 14 infants onto the subspace identified by the first two components of PLS-DA, demonstrating good accuracy. The area under the receiver operating characteristic (ROC) curve, estimated through 1000 repetitions of 5-fold cross-validation, was 0.73.

**Figure 3:**
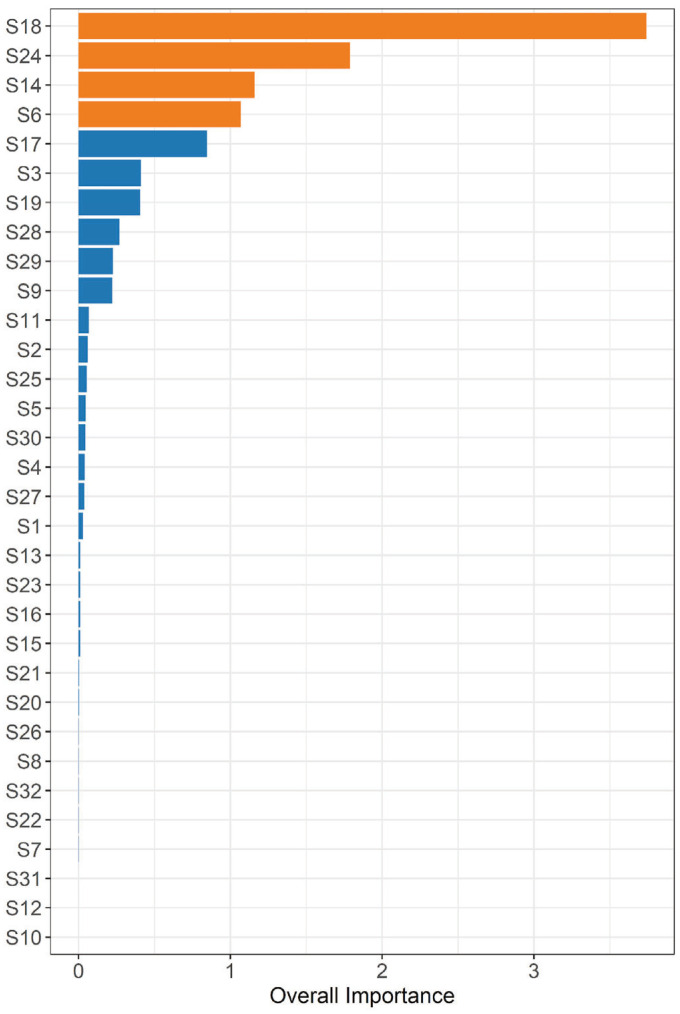
Prediction of bronchopulmonary dysplasia: displaying the variable importance of the 32 e-nose nanosensors as estimated by the gradient boosting machine (GBM). Nanosensors represented by orange bars were statistically significant in exact logistic regression, demonstrating a significant odds ratio.

We further investigated the classification ability (overall importance) of individual nanoprobes using a machine learning predictive algorithm (Figure 3). The most important probe was S18, followed by S24, S14, S6, and S17. Using a logistic model with exact methods, we found that S18, S24, S14, and S6 were significantly associated with the outcome ‘no-BPD’ vs ‘BDP’ (*p* = 0.019, 0.009, 0.043, 0.047, respectively). After applying the Benjamini and Hochberg method for controlling the false discovery rate (FDR) [29], the four nanoprobes remained statistically significant for an FDR of 10%, with an in-sample AUC of 0.87, 0.89, 0.82, and 0.8, respectively.

**Figure 4:**
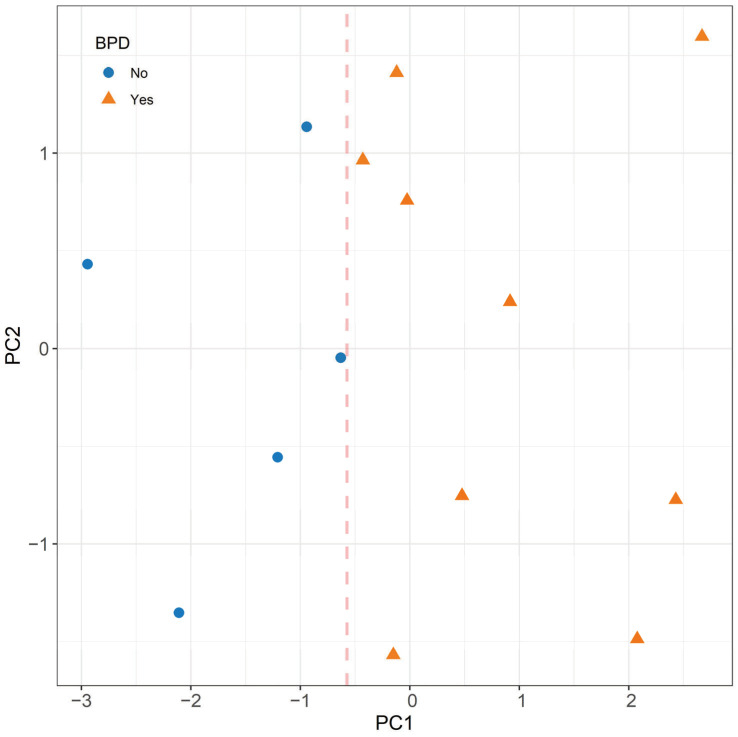
Scatter plot of the first two principal components (PC1 and PC2) resulting from the principal component analysis conducted on the correlation matrix of the 4 significant probes from Figure 3 (S6, S14, S18, and S24). The dashed line represents the discrimination line. PCA is utilized here for purely descriptive and exploratory purposes. Nonetheless, Figure 4 should be interpreted with caution due to the modest sample size and the associated risk of overfitting in PCA.

A principal component analysis was performed on the response patterns of S18, S24, S14, and S6 probes among the 14 preterm infants (Figure 4). The two leading principal components, accounting for 71% of the total variability, are illustrated in Figure 4. The diagram reveals that the first principal component (PC1) enables a perfect separation between infants who are likely and not likely to subsequently develop BPD.

A multivariable classification algorithm based on a random forest was trained with four predictors (S18, S24, S14, and S6), using 1000 trees, one variable considered for splitting at each node (mtry = 1), and a maximum tree depth of three (nodedepth = 3). This random forest model achieved an out-of-bag AUC of 0.82.

We also estimated a penalized linear support vector machine (SVM) with a SCAD penalty [30]. The model was optimized using a grid search across lambda1 values from 0.01 to 1. The procedure was repeated 500 times with different seeds, calculating the selection frequency of each of the 32 enose probes. Among the seven variables with the highest selection frequency, the four most important variables identified by the GBM algorithm (S18, S24, S14, and S6) were consistently selected. By repeatedly splitting the dataset into training (10 cases) and testing (4 cases) sets, we evaluated the out-of-sample AUC, obtaining a median estimate of 0.75 (IQR = 0.67–1).

Lastly, we applied a neural network to the dataset, using five neurons in the hidden layer, a logistic transfer function, weight decay of 0.3, and the BFGS algorithm for optimization [31]. This approach resulted in an out-of-sample AUC estimate of 0.75 (IQR = 0.67–0.83).

## 4. Discussion

To the best of our knowledge, this is the first study to assess VOC profile in EBC using an e-nose in a cohort of very preterm mechanically ventilated infants. The aim was to identify early biomarkers that could predict the subsequent development of bronchopulmonary dysplasia.

The e-nose is an innovative biomimetic technology that simulates the olfactory system to examine VOCs in various organic matrices, including EBC, urine, feces, and blood [32,33].

A variety of technologies have been applied in the study of metabolomic approaches such as gas chromatography-mass spectroscopy (GC-MS), to identify disease-associated VOC metabolites. The e-nose is a cheap, portable instrument for clinical, patient room or field that permits identifying and discriminating between complex mixtures of VOCs without identifying individual chemical species. On the contrary, mass spectrometry methods are highly complex, time consuming, and require sophisticated software and highly qualified staff for the complex interpretations of the analysis [34].

E-noses contain different sensor arrays capable of discriminating between sensor-response patterns of VOCs by using pattern-recognition algorithms [35]. This approach of the e-nose permits a noninvasive early diagnosis of diseases with greatly accelerated prognostics, allowing earlier and more effective treatments, more rapid patient recovery, and shorter less-expensive stays for hospital care. While the e-nose has been utilized as an adjunctive diagnostic tool in the adult population, its application in pediatric patients, especially in non-cooperative patients such as preterm neonates, has been limited [20].

A study conducted by Rogosch et al. describes for the first time the smell prints of VOCs in the tracheal aspirates from preterm with or without bronchopulmonary dysplasia [33]. Moreover, only a few studies evaluated VOCs in blood or feces as potential biomarkers of future BPD in preterms [36].

In pediatrics, the application of e-nose in children with respiratory disease has shown encouraging results. In children with asthma, for example, the e-nose can differentiate between children with asthma and healthy children but also between subjects with uncontrolled symptomatic asthma from asymptomatic controls [37]. Bannier et al. demonstrates also that the e-nose can identify asthma and cystic fibrosis in children with a high feasibility and modest-to-good diagnostic accuracies [38]. Another study conducted by Ferrante et al. demonstrates that a urinary e-nose analysis correlates with airway inflammation in children with asthma [39].

In the current study, we found that VOC profiles in exhaled breath condensate hold the potential for predicting the subsequent development of BPD. An analysis of EBC from babies who will develop BPD, compared to those who will not, revealed that the e-nose device enabled us to discriminate between the two groups soon after birth. VOCs were analyzed within the first 24 h of life and four probes of e-nose showed a strong predictive ability of BPD with an AUC above 0.8. Currently, the primary BPD prediction tool employed in clinical practice relies solely on clinical variables such as gestational age, birth weight, ethnicity, sex, respiratory support, and a fraction of inspired oxygen. It is noteworthy that the predictive performance of this tool improves with advancing postnatal age, with the AUC rising from 0.79 on day 1 to 0.85 on day 28 [40].

Although our findings are preliminary and require replication in a larger cohort, the analysis using e-nose suggests the potential for the improved prediction of BPD starting from the first day of life. Moreover, the non-invasive and cost-effective nature of e-nose analysis positions it as a promising biomarker for later pulmonary disease. Notably, none of the previously studied biomarkers were effectively implemented in clinical practice to identify children at risk of developing moderate to severe BPD [41].

We speculate that serial analyses in the first days of life of VOC profile by e-nose could further increase the predictive ability of BPD; the latter should be evaluated in a future study.

Great interest lies in the identification and validation of early biomarkers that can predict BPD development, since it could offer the potential to reduce health-care costs. Early diagnosis would allow for the initiation of therapies during the early stages of BPD, when there is a therapeutic window of opportunity [42]. Furthermore, it would help avoid unnecessary treatments and their associated risks in infants who are not at risk of developing BPD [43,44].

Our study is subject to certain limitations.

First, due to the specific patient population, we conducted a pilot study with a limited sample size. Recruiting extremely low gestational age (≤31 weeks) infants poses significant challenges due to their rarity, the need for stringent inclusion criteria, ethical considerations, and the fragile health of the infants, which limits their participation in non-essential studies. While this may impact the generalizability of our results, it is important to emphasize that this is an exploratory study aimed at evaluating the potential of the e-nose in predicting BPD, not building a prediction tool for immediate clinical use. The findings provide preliminary insights that should be validated in larger cohorts and linked to inflammatory markers to strengthen their significance, guiding future studies. To ensure the robustness of our findings, we implemented cross validation across all data analyses to mitigate the risk of overfitting. This statistical approach provides an honest and not overly optimistic assessment

Second, this study did not incorporate additional chemical analytical techniques, such as gas chromatography-mass spectrometry (GC-MS), which would allow the identification of specific VOCs associated with BPD. Integrating such methods would be a significant advancement in understanding the diverse pathways involved in lung injury pathophysiology and distinguishing between different forms of BPD, often referred to as BPD endotypes. This deeper understanding could pave the way for the development of personalized therapeutic strategies tailored to specific patient needs [45].

## 5. Conclusions

In conclusion, the e-nose is a user-friendly, handheld electronic device that enables non-invasive and rapid breath sampling. It holds potential as a novel clinical tool for predicting BPD in preterm neonates. Identifying VOCs in BPD patients could enhance our understanding of BPD pathophysiology, and these early diagnostic biomarkers show promise for the development of personalized and tailored treatments within the therapeutic window of opportunity.

## Figures and Tables

**Table 1:** Demographic and clinical characteristics of preterm infants, distinguishing between those who subsequently developed bronchopulmonary dysplasia (BPD) and those who did not. Means and standard deviations describe continuous variables, frequencies, and percentages for categorical variables.

Variable	No-BPD (*n* = 5)	BPD (*n* = 9)	*p*-Value
Gestational age, weeks	28.1 ± 2.9	25.9 ± 1.8	0.1
Sex (female)	3 (60)	6 (67)	1
Birth weight, grams	1182 ± 447	894 ± 281	0.2
Apgar 5 min	7 ± 1.7	7.6 ± 1.1	0.5
Surfactant	5 (100)	9 (100)	-
Pulmonary hypertension	0 (0)	0 (0)	-
Cesarean section	3 (60)	5 (56)	1
NO supplementation	0 (0)	0 (0)	-
Antibiotics therapy (ampicillin+gentamicin)	5 (100)	9 (100)	-
Infectious risk (chorioamnionitis)	0 (0)	2 (22)	0.5
Pathological course of pregnancy (gestured)	3 (60)	3 (33)	0.6

## Data Availability

Data are contained within the article.
